# *Bacillus cereus* extracellular vesicles act as shuttles for biologically active multicomponent enterotoxins

**DOI:** 10.1186/s12964-023-01132-1

**Published:** 2023-05-15

**Authors:** Tanja Buchacher, Astrid Digruber, Markus Kranzler, Giorgia Del Favero, Monika Ehling-Schulz

**Affiliations:** 1 Functional Microbiology, Institute of Microbiology, Department of Pathobiology, University of Veterinary Medicine, Vienna, Austria; 2 Department of Food Chemistry and Toxicology, Faculty of Chemistry, University of Vienna, Vienna, Austria; 3 Core Facility Multimodal Imaging, Faculty of Chemistry, University of Vienna, Vienna, Austria

**Keywords:** *Bacillus cereus*, Extracellular vesicles, Host–pathogen interaction, Multicomponent toxin, Non-hemolytic enterotoxin, SMase, 3D-SIM microscopy

## Abstract

**Background:**

Extracellular vesicles (EVs) from Gram-positive bacteria have gained considerable importance as a novel transport system of virulence factors in host–pathogen interactions. *Bacillus cereus* is a Gram-positive human pathogen, causing gastrointestinal toxemia as well as local and systemic infections. The pathogenicity of enteropathogenic *B. cereus* has been linked to a collection of virulence factors and exotoxins. Nevertheless, the exact mechanism of virulence factor secretion and delivery to target cells is poorly understood.

**Results:**

Here, we investigate the production and characterization of enterotoxin-associated EVs from the enteropathogenic *B. cereus* strain NVH0075-95 by using a proteomics approach and studied their interaction with human host cells in vitro. For the first time, comprehensive analyses of *B. cereus* EV proteins revealed virulence-associated factors, such as sphingomyelinase, phospholipase C, and the three-component enterotoxin Nhe. The detection of Nhe subunits was confirmed by immunoblotting, showing that the low abundant subunit NheC was exclusively detected in EVs as compared to vesicle-free supernatant. Cholesterol-dependent fusion and predominantly dynamin-mediated endocytosis of *B. cereus* EVs with the plasma membrane of intestinal epithelial Caco2 cells represent entry routes for delivery of Nhe components to host cells, which was assessed by confocal microscopy and finally led to delayed cytotoxicity. Furthermore, we could show that *B. cereus* EVs elicit an inflammatory response in human monocytes and contribute to erythrocyte lysis via a cooperative interaction of enterotoxin Nhe and sphingomyelinase.

**Conclusion:**

Our results provide insights into the interaction of EVs from *B. cereus* with human host cells and add a new layer of complexity to our understanding of multicomponent enterotoxin assembly, offering new opportunities to decipher molecular processes involved in disease development.

Video Abstract

**Supplementary Information:**

The online version contains supplementary material available at 10.1186/s12964-023-01132-1.

## Introduction

The endospore-forming *Bacillus cereus* is a Gram-positive, food poisoning-associated pathogen, with the ability to cause severe gastrointestinal tract infections [1, 2]. Besides, there is a growing body of evidence supporting the association between *B. cereus* infections and a range of acute non-gastrointestinal tract diseases, such as sepsis and infections of the central nervous system, particularly in immunosuppressed patients and newborns [3, 4]. The pathogenesis of *B. cereus* is mainly related to the heat- and gastric acid-stable emetic toxin cereulide, a cyclic dodecadepsipeptide causing vomiting and – in severe cases – organ failure [5, 6], while the multicomponent pore-forming enterotoxins, such as non-hemolytic enterotoxin (Nhe) and hemolysin BL (Hbl), provoke a diarrheal syndrome [7–10].

The tripartite enterotoxin Nhe is present in almost all enteropathogenic *B. cereus* strains [1, 7, 11, 12] however, the exact mode of action of the Nhe toxin at the cellular level is still poorly understood. Several studies have shown that all three toxin components NheA, NheB and NheC are necessary for optimal pore formation and, finally, cell membrane leakage in vitro, which requires a specific concentration, ratio and binding order of the Nhe components at the target cell surface [13–15]. NheC has been suggested to be mandatory in the priming step, however, due to its low abundance and the lack of tailored analytical tools, its detection remains challenging [14, 16]. Only few proteomics studies have detected NheC in the exoproteome of *B. cereus* [17, 18]. In addition, several other exoproteins, including proteases and membrane-damaging phospholipases, have been discussed recently as putative virulence factors in *B. cereus* pathogenicity [19–21]. The sphingomyelinase (SMase) of *B. cereus* has been shown to synergistically interact with Nhe as well as with Hbl, suggesting its contribution to the severity of the disease [22, 23]. *B. cereus* SMase, similar to other bacterial SMases is able to hydrolyze sphingomyelin [24], thereby affecting the dynamics of membranes and the host immune system. Recently, it has been demonstrated that toxicity of a given strain correlates with the quantity of secreted SMase, the B component of Nhe and proteolytic activity [25]. Nevertheless, how multicomponent enterotoxins are transported in the extracellular milieu and finally delivered to target host cells remains poorly understood.

In the past years, naturally produced extracellular vesicles (EVs) from bacteria have gained considerable importance as a novel transport system of multiple virulence factors in host–pathogen interaction and pathogenesis. EVs represent spherical membrane-enclosing structures that are released as a conserved mechanism for cell-free inter- and intra-species cellular communications across all three domains of life [26, 27]. Bacterial membrane vesicles have been extensively studied in Gram-negative bacteria [28, 29], however, recent studies have also demonstrated the production of EVs in Gram-positive bacteria [30, 31]. Although the exact mechanisms of vesicle biogenesis and transport through the thick peptidoglycan layer of Gram-positive bacteria remains poorly understood, a possible mechanism has been described by the activity of cell wall degrading enzymes which generate holes in the peptidoglycan layer and allow the release of EVs into the surrounding [32–35]. Bacteria-derived EVs are loaded with a large diversity of bioactive compounds, including proteins, nucleic acids, and virulence factors [30, 36]. The cargo of EVs determines their biological functions, ranging from bacterial survival, biofilm formation, resistance to antibiotics, host immune invasion and modulation, and infection [31, 37].

EVs from pathogenic Gram-positive species carry a range of toxins and molecules that are involved in immune evasion [30]. In *Staphylococcus aureus*, EVs have been shown to deliver virulence-associated factors, causing cytotoxicity to host cells [38, 39]. EVs associated with cytosolic pore-forming toxins of *Streptococcus pneumoniae* have been shown to bind complement proteins, thereby promoting pneumococcal evasion of complement-mediated opsonophagocytosis [40]. Pneumococcal vesicles are also able to induce protection against infection in vivo [41], while some studies revealed their contribution to inflammatory responses and tissue damage in hosts [42, 43]. Likewise, *B. anthracis*-derived EVs contain biologically active anthrax toxin components that are toxic to macrophages and induce a protective response in immunized mice [44].

To elucidate the role of EVs in the pathogenesis of enteropathogenic *B. cereus*, we characterized their production and cargo content by using a proteomics approach and studied their interaction with human host cells in vitro. Our study provides evidence that *B. cereus* EVs are loaded with several virulence-associated factors, such as SMase, phospholipase C, and the multicomponent enterotoxin Nhe. We could show that *B. cereus* EVs interact with intestinal epithelial cells via cholesterol-rich domains and dynamin-mediated endocytosis, leading to Nhe internalization and delayed cytotoxicity. Notably, SMase packed in *B. cereus* vesicles complemented Nhe-induced hemolysis in vitro, highlighting the function of EVs as vehicle of multiple virulence factors for their concerted actions on host cells. The identification of toxin-loaded EVs in *B. cereus* adds a new layer of complexity to our understanding of how multicomponent enterotoxins are assembling and further affecting host interaction and pathogenesis.

## Results

### *B. cereus* secretes EVs into the culture supernatant, containing the multicomponent enterotoxin Nhe and the membrane active enzyme SMase

**Fig. 1:**
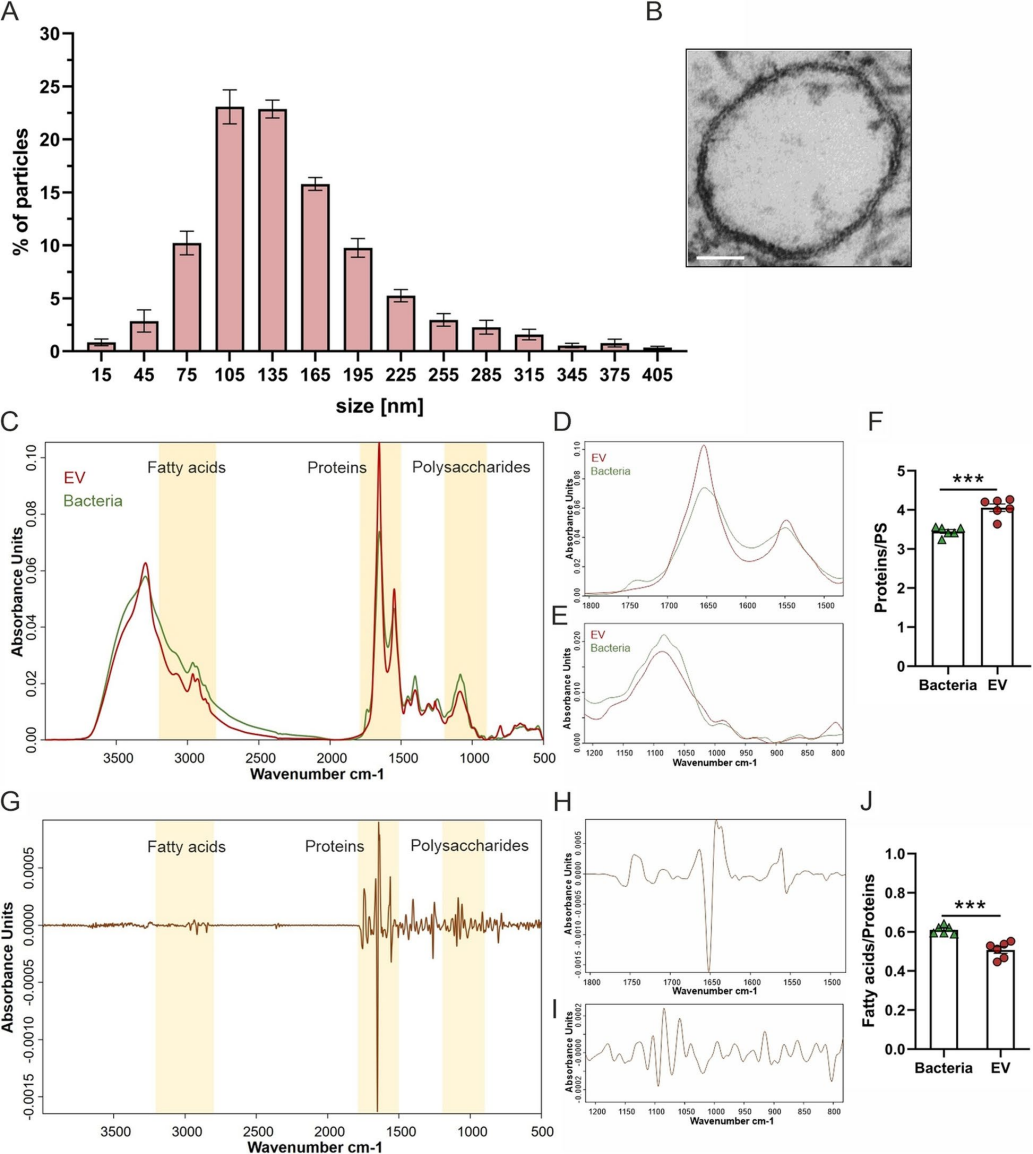
*B. cereus* releases EVs in vitro. EVs of *B. cereus* NVH0095-75 were characterized using nanoparticle tracking analysis (NTA), transmission electron microscopy and Fourier transform infrared (FTIR) spectroscopy. **A** Purified EVs from three biological independent replicates (three technical measurements each) were subjected to NTA for analysis of size distribution. **B** Resin-embedded TEM, used to determine the shape and confirm the size, revealed round-shaped EVs from *B. cereus* (scale bar 50 nm). **C-I** FTIR was used to generate metabolite fingerprints from isolated *B. cereus* EVs and intact bacterial cells. EVs isolated from six independent bacterial cultures were analyzed by FTIR. Representative full-range normalized spectra are shown (**C**), highlighting the spectral regions used for subsequent chemometric analysis. **D** and **E** provide a zoom-in into the region characteristic for proteins (1720–1500 cm-1) and polysaccharides (1200–900 cm-1), respectively. **G** As revealed by 2^nd^ derivative subtraction spectral analysis of EVs versus bacterial cells, spectral differences between EVs and bacteria were most pronounced in the spectral regions accounting for proteins (zoom-in see **H**) and polysaccharides (zoom-in see **I**). **F** and **H** Calculation of spectroscopic ratios of highlighted regions in (**C**) as described in the method section. Statistical significance is calculated using a two-tailed Student’s t-test (***p < 0.001)

In recent years, extracellular prokaryotic membrane vesicles have been reported to play an important role for Gram-negative bacteria in cell–cell communication and transport of virulence factors to host cells [30], but still little is known about their role in Gram-positive bacteria. With the aim to evaluate the production and secretion of spherical EVs from Gram-positive *B. cereus*, the enteropathogenic strain NVH0075-95 was grown in LB broth for 17 h at 30 °C and pelleted vesicles were visualized by transmission electron microscopy (TEM). TEM exhibited intact spherical structures with a diameter of up to 200 nm, suggestive of extracellular membrane vesicles (Fig. 1B). The size distribution of vesicles was confirmed by nanoparticle tracking analysis (NTA) measurements (Fig. 1A), revealing *B. cereus* EVs with a peak size ranging from 75 to 200 nm in diameter. Both complementary approaches highlighted the presence of *B. cereus* EVs.

These results strongly support the notion that Gram-positive *B. cereus* actively produces and secretes EVs, heterogeneous in size, into the extracellular milieu during cell growth in vitro.

Fourier transform infrared (FTIR) spectroscopy was employed to further characterize the *B. cereus* EVs and determine their lipid and protein content. Spectra of isolated EVs were recorded in the spectral range of 4000 to 500 cm^−1^ and preprocessed spectra were subjected to chemometric analysis. In parallel, spectra were recorded from bacteria to determine the difference in the spectral profiles of EVs and bacteria (Fig. 1C-E). As revealed by subtraction analysis of the 2nd derivate spectra, (Fig. 1G) the most prominent differences between EVs and bacteria were found in the protein region (in amid I and amid II bands (1700–1600 cm^−1^ and 1600–1500 cm^−1^, respectively), indicating changes in peptide backbones (Fig. 1G, H) and the polysaccharides region (1200–900 cm^−1^). The latter region includes functional groups in polysaccharides of cell walls, phosphate-containing molecules and cell surface glycostructures (Fig. 1G, I). To gain further insights into the spectral differences between bacteria and their EVs, we calculated the ratios of proteins to polysaccharides and fatty acids to proteins. Compared to bacterial cells, the ratio of proteins to polysaccharides was significantly higher in EVs (Fig. 1F), whereas the ratio of fatty acids to proteins was higher in bacteria than in EVs (Fig. 1J). These results indicate that EVs differ in their protein/fatty acid composition from bacteria, which is reflected in their characteristic spectral fingerprints. Overall, FTIR spectroscopy proved to be a suitable method for determining *B. cereus*-specific EV fingerprints, which could be used to monitor the quality of EVs.

**Fig. 2:**
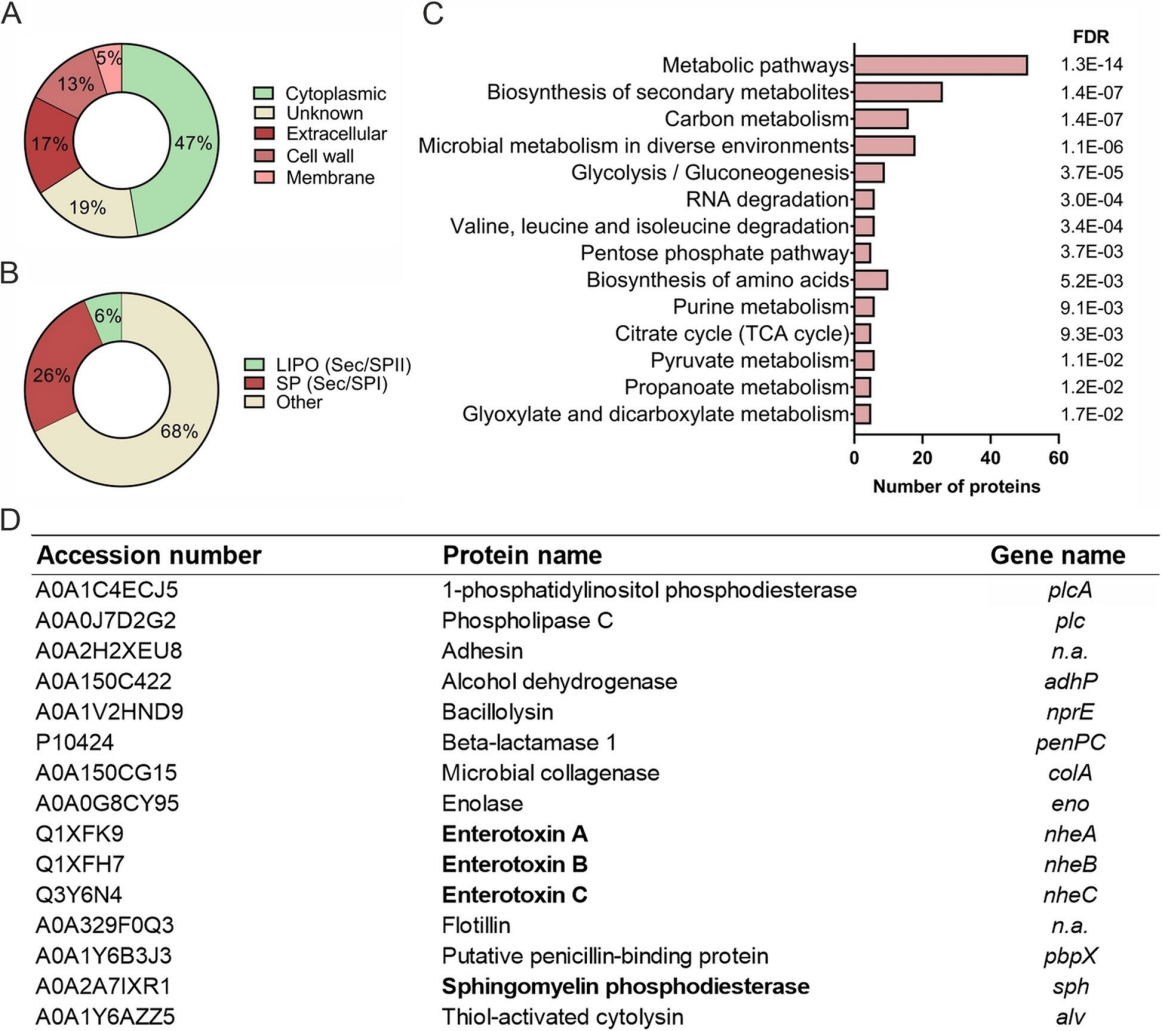
Proteomic profiling of *B. cereus* extracellular vesicles revealed several enterotoxins. The protein cargo of *B. cereus* secreted extracellular vesicles was determined by LC–MS/MS. Proteins commonly identified in two biologically independent experiments were included in the proteomic analysis. **A** Subcellular localization of EV proteins predicted with the aid of PsortB and illustrated in proportional numbers. **B** Prediction of the number of proteins with a secretory signal peptide, using SignalP. **C** KEGG pathway enrichment analysis of EV proteins. Pathways with a false discovery rate (FDR) < 0.05 were considered as significantly enriched. **D** Overview of virulence-associated factors identified in *B. cereus* NVH0095-75 EVs

Since virulence factors form a large constituent of the protein content in bacteria-derived EVs [30, 39], we next characterized the cargo proteins of the *B. cereus* EVs by liquid chromatography–tandem mass spectrometry (LC–MS/MS) to screen for virulence factors. The majority of identified proteins (Additional file 3: Table 1) were predicted to be cytoplasmic (47%) and membrane- or cell wall-associated (5% and 13%, respectively), while 17% of the proteins were predicted to occur extracellularly (Fig. 2A, Additional file 4: Table 2). Based on SignalP analysis, 26% of proteins in EVs contain a Sec signal peptide, predicted to be secreted via the classical secretory pathway. In contrast, 68% of the proteins have no predicted signal peptide, indicating that the release of EVs appears to be an important process for the secretion of proteins.

Only 6% are predicted lipoproteins, which have a signal peptidase II cleavage site and primarily belong to the ATP-binding cassette (ABC) transporter substrate-binding proteins (Fig. 2B, Additional file 1: Figure S1, Additional file 5: Table 3). Pathway enrichment analysis on identified proteins revealed various biological processes to be overrepresented in EVs, including metabolic pathways and biosynthesis of secondary metabolites (Fig. 2C). Among the identified EV proteins, we found all three components of the enterotoxin complex Nhe, which plays a key role in *B. cereus* enteropathogenicity [1, 2], and SMase, which hydrolyzes sphingomyelin and was reported to be a crucial factor for the toxicity of a given strain [25]. Moreover, several virulence-associated factors were identified in *B. cereus*-derived EVs (Fig. 2D), including penicillin-binding protein (PBP), adhesin, enolase, collagenase, alcohol dehydrogenase-like protein, bacillolysin, thiol-activated cytolysin and phospholipase C (PLC).

### *B. cereus* EVs deliver the multicomponent enterotoxin Nhe to human intestinal Caco2 cells

**Fig. 3:**
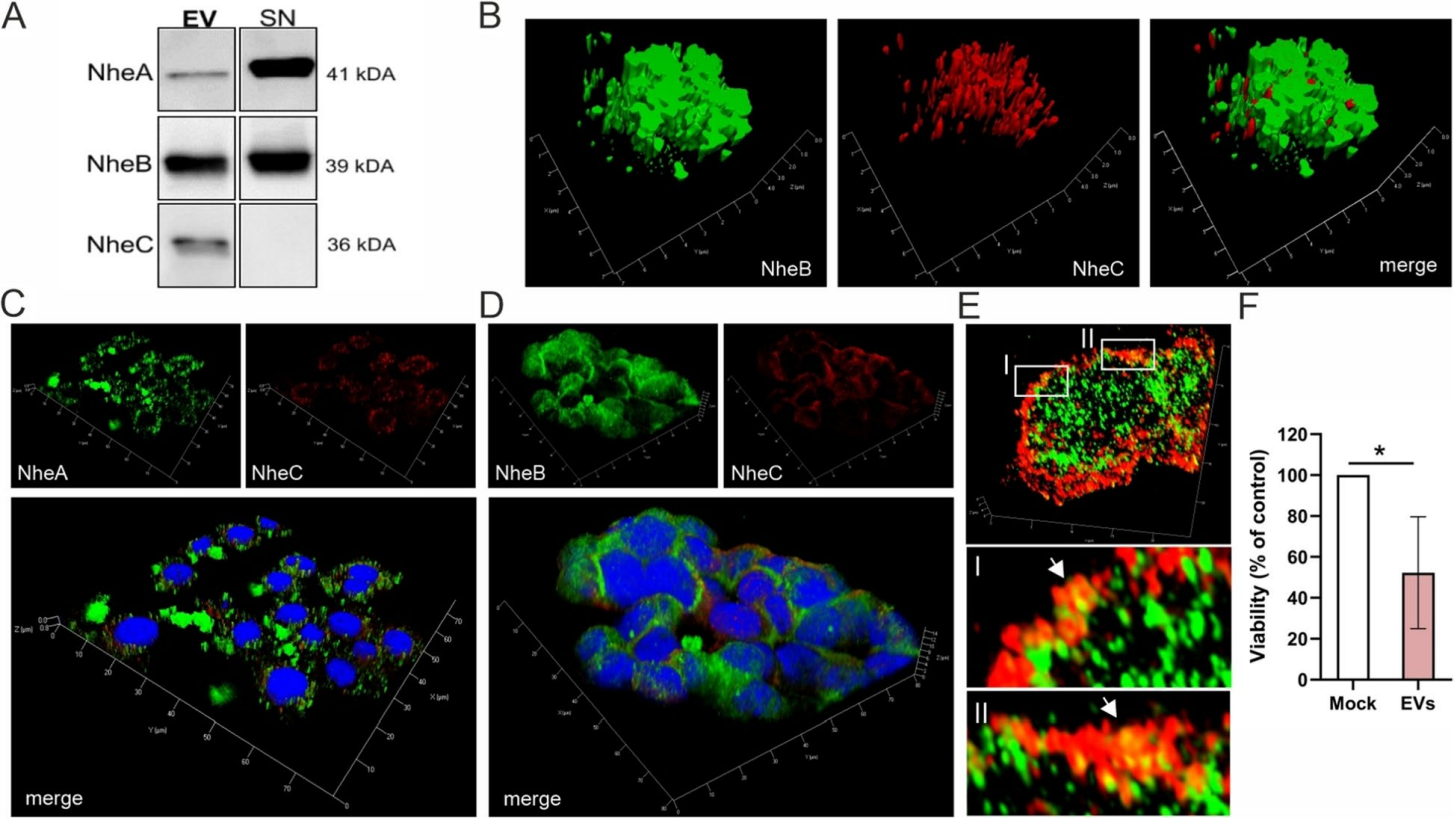
The multicomponent enterotoxin Nhe is packed in *B. cereus* vesicles and transported through vesicles to human intestinal Caco2 cell. Mouse monoclonal antibodies (anti-NheA IgG1, anti-NheB IgG1κ), anti-NheC IgM) were used for the specific detection of the Nhe components [80]. **A** As shown by immunoblot analyses, NheA and NheB are detectable in *B. cereus*-derived extracellular vesicles (EV) and vesicle-free supernatant (SN) while NheC is only detectable in EV. **B** The presence of NheB (AF488, green) and NheC (AF568, red) in *B. cereus* vesicle aggregates was confirmed with 3D-SIM microscopy. **C-E** To study the delivery of Nhe enterotoxin to human intestinal Caco2 cells, cells were treated with 200 µg/mL of *B. cereus* EVs for 2 h followed by several washing steps. **C** NheA (AF568; red), NheB (AF488; green) and cell nuclei (DAPI; blue), or (**D**) NheB (AF488; green), NheC (AF568; red) and cell nuclei (DAPI; blue) were visualized by 3D-SIM microscopy. **E** 3D-SIM fluorescent images at single cell level showed colocalization of NheB (AF488, green) and NheC (AF568, red) components (I, II) at the edges of Caco2 cells. **F** Viability of Caco2 cells stimulated with EVs was determined after 24 h using the Vita-Orange Cell Viability Reagent and expressed as a percentage with respect to untreated control cells for three independent biological experiments (mean ± standard error of the mean (SEM). Statistical significance is calculated using two-tailed Student’s t-test (*p < 0.05)

Since the cytotoxic strain NVH0075-95 expresses the tripartite pore-forming toxin Nhe but lacks the *hbl* genes [11, 25], we further investigated the association of the Nhe enterotoxin with EVs. Secreted and purified vesicles from *B. cereus* NVH0075-95 were analyzed by using highly specific monoclonal antibodies for each Nhe component. Western immunoblotting confirmed the presence of the three single components in EVs. Notably, NheC was highly concentrated in *B. cereus* EVs, while it could not be detected in the vesicle-free supernatant, even when eightfold amounts of proteins were loaded. Both components NheB and predominantly NheC were packaged in vesicles, whereas NheA was detected in vesicles but mainly located in the vesicle-free supernatant (Fig. 3A). Three-dimensional structured illumination microscopy (3D-SIM) of *B. cereus* EVs confirmed the co-localization of enterotoxin components NheB and NheC (Fig. 3B), as well as NheA and NheC (Additional file 2: Figure S2A).

Given the important role of the host gastrointestinal tract in the pathogenesis of *B. cereus* [45], we next studied the delivery of *B. cereus* vesicle-associated Nhe components to human intestinal Caco2 cells, followed by cellular internalization using 3D-SIM. The three Nhe subunits were detected in Caco2 cells already at 2 h upon treatment with *B. cereus* EVs (Fig. 3C-D). At 2 h post-stimulation with *B. cereus* vesicles, the morphology of Caco2 cells remained unchanged and no nuclear fragmentation was observed when compared to untreated Caco2 cells (Fig. 3C-D, Additional file 2: Figure S2B).

Moreover, 3D-SIM images at single cell level showed colocalization of NheB and NheC components at the edges of Caco2 cells (Fig. 3E, insert I, II). These findings are consistent with the hypothesis that the vesicle cargo containing Nhe components was being internalized into intact Caco2 cells. In order to determine whether *B. cereus*-derived EVs, enriched with virulence factors (see Fig. 2D), induce cytotoxicity, and thus play a critical role in microbial pathogenesis, Caco2 cells were treated with EVs for 24 h. At 24 h post-stimulation, *B. cereus*-derived EVs induced a cytotoxic effect with a cell viability of less than 50% (Fig. 3F). These results suggest that the delivery of EV cargo to Caco2 cells is a prerequisite for cytotoxicity.

### Cellular uptake of *B. cereus* extracellular vesicles is mediated via cholesterol-rich microdomains and endocytosis

**Fig. 4:**
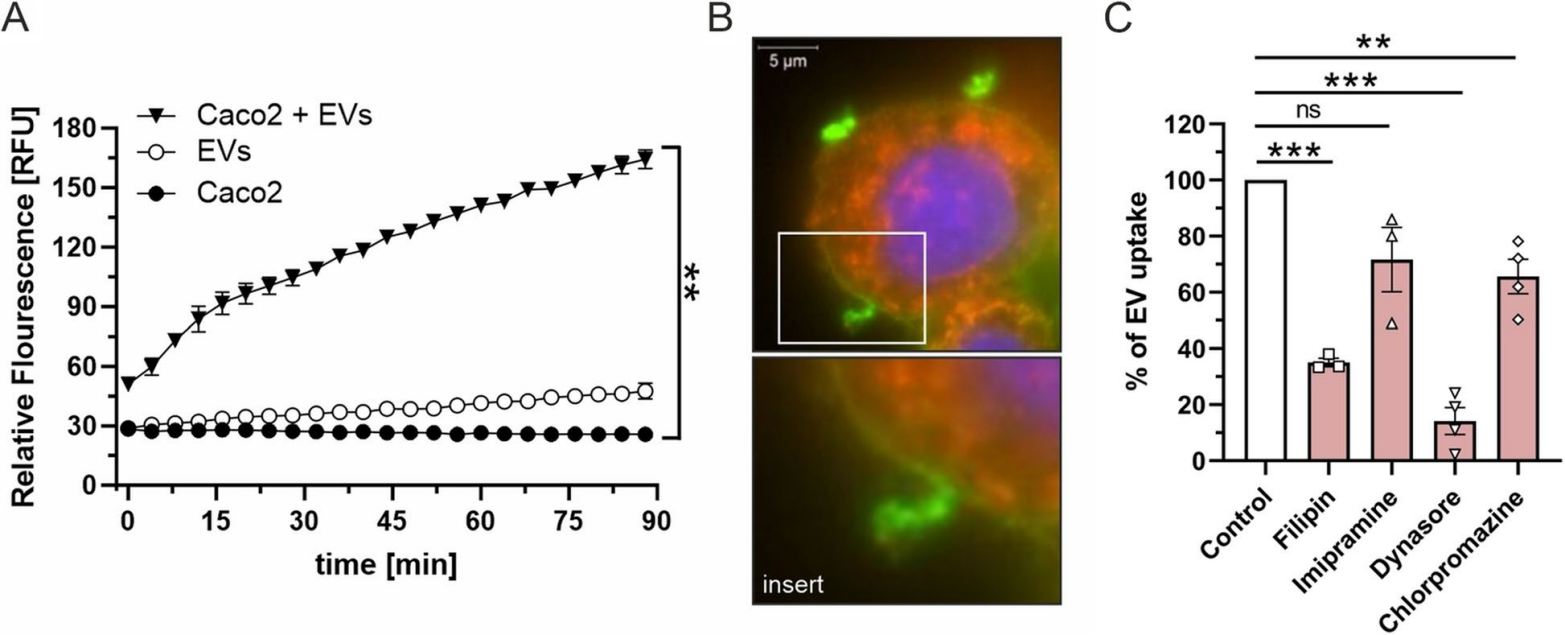
Cellular uptake of *B. cereus* extracellular vesicles is mediated by means of membrane fusion via cholesterol-rich domains and endocytosis. **A** For *B. cereus* EV uptake, Rhodamine-R18 labeled *B. cereus* EVs (5 µg) were applied to Caco2 cells and the fluorescence intensity was measured every two minutes (shown here every six minutes for the sake of clarity) up to 90 min at 37 °C using a microtiter reader. An increase in fluorescence intensity indicates membrane fusion, shown as mean ± standard error of the mean (SEM) for three independent biological experiments. **B** Vesicles containing NheB were detected with AF488 (green) and found to be aggregated to the edges and surfaces of Caco2 cells indicating fusion (scale bar 5 µm, 3D-SIM images). **C** Inhibition of vesicle uptake by Caco2 was studied in the presence of either cholesterol-sequestering agents Filipin III (10 µg/ml) and Imipramine (10 mM), or dynamin and clathrin-mediated endocytosis inhibitors Dynasore (80 μM), and chlorpromazine (15 ug/ml; all from Sigma Aldrich, USA), respectively. After 1 h of cell treatment with the inhibitors, EVs were added and cultured for 90 min. The percentage of EV uptake in the presence of inhibitors was normalized to internalization in untreated cells. Mean ± standard error of the mean (SEM) is shown for three or four independent biological experiments. Statistical significance is calculated using two-tailed Student’s t-test (**p < 0.01, ***p < 0.001)

Since we demonstrated that *B. cereus* uses EVs to transport the tripartite enterotoxin Nhe into host cells, we next investigate whether *B. cereus* vesicles are able to enter human intestinal epithelial cells via membrane fusion to release their contents. To this end, EVs of *B. cereus* were fluorescently labeled with the lipophilic dye octadecyl rhodamine B chloride (R18) and subsequently applied to intact Caco2 cells. R18 fluorescence is quenched at high concentrations in cell membranes and dequenched when diluted by fusion with the host cell membrane. Thus, an increase in R18 fluorescence directly correlates with the fusion reaction of the host cell membrane and vesicles. Interaction of R18-labeled NVH0075-95 EVs with Caco2 cells showed a rapid and significantly time-dependent increase in fluorescence, indicating membrane fusion of vesicles and host cells (Fig. 4A). In contrast, the fluorescence did not increase in control samples containing only Caco2 cells or only rhodamine-R18 labeled vesicles. Moreover, confocal microscopy indicated the attachment and aggregation of NheB-loaded vesicles to the edges and surfaces of Caco2 cells (Fig. 4B) with a partial distribution of NheB along the cell membrane (Fig. 4B, insert). To identify the entry route of EVs, host cells were treated with rhodamine-R18 labeled vesicles in the presence of chemical drugs to block cellular functions. The cholesterol sequestering agent Filipin III complex, which binds to cholesterol in cholesterol-rich microdomains in the cell membrane, significantly reduced the uptake of *B. cereus* vesicles by approx. 65% as compared to internalization by untreated Caco2 cells (Fig. 4C, Additional file 2: Figure S2C).

Moreover, EV uptake could be significantly blocked by using clathrin- and dynamin-mediated endocytosis inhibitors, Chlorpromazine and Dynasore, respectively. In the presence of Chlorpromazine, EV internalization was significantly reduced by 29% compared to control, (Fig. 4C, Additional file 2: Figure S2D), whereas treatment with Dynasore revealed the strongest decrease (by 82%) in EV uptake (Fig. 4C; Additional file 2: Figure S2D). By comparison, Imipramin, a substance with surfactant properties and known for blocking acidic sphingomyelinase [46], showed a slight, but not significant decrease in vesicle internalization (by 28%) (Fig. 4C, Additional file 2: Figure S2D). Amilorid and Cytochalasin D, inhibitors blocking macropinocytosis and F-actin polymerization, respectively, showed no effect on *B. cereus* vesicle internalization by Caco2 cells (data not shown).

Collectively, these results indicate that the uptake of *B. cereus* vesicles by Caco2 cells occurs through multiple pathways but predominantly via cholesterol-rich domains and dynamin-mediated endocytosis.

### Bioactive *B. cereus* vesicles induce hemolysis and elicit an inflammatory immune response in human blood cells

**Fig. 5:**
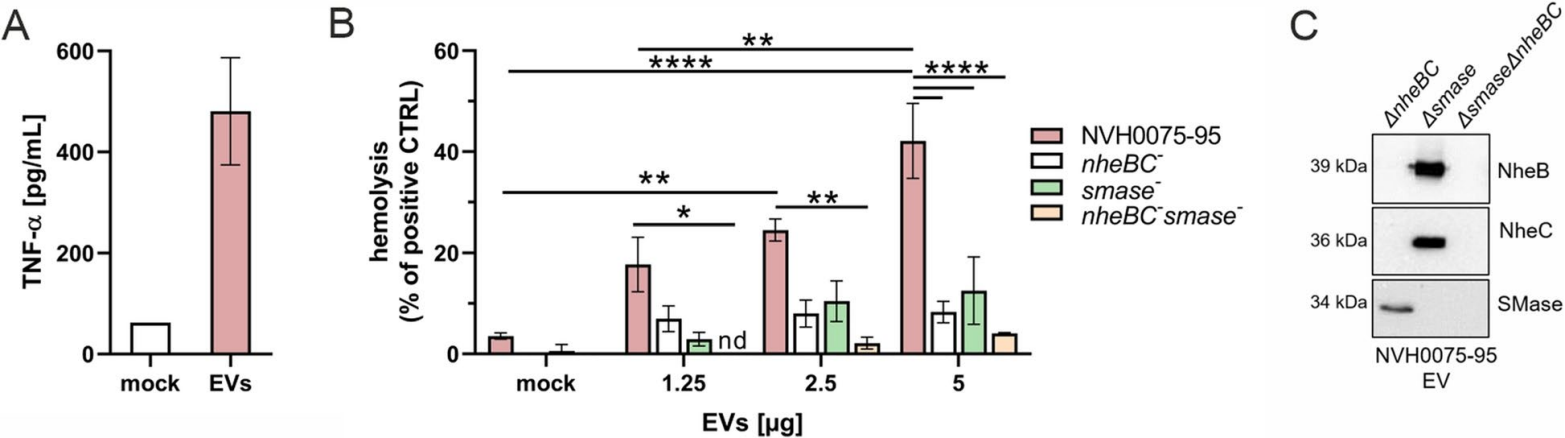
Hemolytic and proinflammatory activity of *B. cereus* extracellular vesicles. **A** Human monocytes (2.5 × 10^6^ cells/mL) were stimulated with 100 µg/mL of EV for 4 h at 37 °C / 5% CO_2_ and pro-inflammatory cytokine secretion was assessed using ELISA. Mock stimulation served as control. Concentrations are expressed as mean ± standard error of the mean (SEM) for three independent biological experiments. **B** Human erythrocytes were stimulated with different concentrations of EVs from wild type strain NVH0075-95 and EVs derived from single *∆nheBC*, single *∆smase* and double *∆nheBC∆smase* mutants. After 1 h of stimulation at 37 °C, 5% CO_2,_ erythrocytes were pelleted and the supernatant was transferred into a 96-well microtiter plate. The absorbance was measured at 540 nm using a microplate reader. The hemolytic activity is calculated as percentage of the positive control Triton X-100 and expressed as mean ± standard error of the mean (SEM) for three independent biological experiments. Statistical significance is calculated using the two-way ANOVA with Tukey's multiple comparisons test (*p < 0.05, **p < 0.01, ****p < 0.0001). **C** Single or double deletion of *nheBC* and *smase* in EVs was confirmed by western blotting

An important facet of prokaryotic EVs is their immunoreactivity via triggering pro-inflammatory immune responses that likely impact pathogenesis [40, 47]. In our model, *B. cereus* EVs triggered the secretion of the pro-inflammatory cytokine tumor necrosis factor-alpha (TNF-α) in human primary monocytes (Fig. 5A). TNF-α levels were highly upregulated at 4 h post-stimulation with *B. cereus* vesicles, indicating the onset of an inflammatory response.

To evaluate whether toxin-containing EVs from *B. cereus* NVH0075-95 are biologically active, we assessed their hemolytic activity on human erythrocytes. *B. cereus* EVs caused hemolysis in a concentration-dependent manner (Fig. 5B). Next, we generated EVs from an NVH0075-95 NheBC null mutant (*ΔnheBC)* to investigate the contribution of Nhe to EV-induced hemolysis. The successful deletion of *nheBC* was confirmed by immunoblot analysis (Fig. 5C). Notably, the hemolytic activity of EVs from the isogenic *ΔnheBC* mutant was clearly reduced compared with EVs originating from the wild-type strain. However, the *ΔnheBC* deletion did not completely abolish hemolysis (Fig. 5B), suggesting the involvement of other EV-derived virulence factors. Since SMase, a host damaging phospholipase shown to synergistically interact with Nhe in vitro and in vivo [23], was identified in NVH0075-95 EVs by proteome profiling (Fig. 2D), we next generated EVs from an NVH0075-95 *Δsmase* mutant and a double knockout mutant of *nheBC* and *smase* (*ΔsmaseΔnheBC*). The EVs derived from the *Δsmase* mutant showed decreased hemolytic activity to the same extent as *ΔnheBC* mutant vesicles. However, EVs originating from the double depletion of SMase and NheBC (*ΔsmaseΔnheBC*) abolished hemolysis (Fig. 5B), emphasizing the pivotal role of EVs as vehicles for a cooperative action of enterotoxin Nhe and phospholipase SMase in *B. cereus* pathogenicity.

## Discussion

EVs secreted by clinically relevant bacteria are considered important mediators of host–pathogen interactions. In recent years, there is growing evidence that pathogenic Gram-positive bacteria deliver multiple virulence factors via EVs in a protected manner to target host cells and thereby contribute to bacterial pathogenesis [30, 31]. The Gram-positive endospore-forming pathogen *B. cereus* secretes a wide variety of membrane-damaging toxins that could act together or synergistically with each other and other virulence factors to enhance the cytotoxic potential [22, 23, 25]. However, the exact mechanism of *B. cereus* toxin and virulence factor delivery to the host and their uptake into target cells is hitherto unknown.

Here we show that the enteropathogenic *B. cereus* NVH0075-95 secretes biologically active EVs, loaded with exotoxins and various virulence factors, which elicit an inflammatory immune response in host cells. In line with data from *B. anthracis* [44], a close relative of *B. cereus* [1, 2], *B. cereus* EVs revealed spherical-like structures with an average size of 150 nm confirmed by light scattering and TEM analysis. Similar sizes were detected in other Gram-positive bacteria [48, 49], suggesting a natural, usual common size of Gram-positive bacteria-derived EVs.

Proteome profiling of *B. cereus* EVs revealed that proteins derived from the cytoplasm represent the most abundant EV component. Similar observations were reported for other Gram-positive bacteria, such as *B. anthracis, Enterococcus faecalis, S. aureus and S. pneumoniae* [39, 41, 44, 47, 50, 51]. Since cytoplasmic proteins lack secretion signals and more than half of the proteins detected in *B. cereus* vesicles do not have a predicted signal peptide, it is reasonable to conclude that EVs represent a specific secretory mechanism to *B. cereus*, as lately reported for *S. aureus* [51]. Several virulence-associated factors were identified in *B. cereus* EVs, including the multicomponent enterotoxin Nhe as well as membrane-active enzymes, such as the phospholipase PLC [52] and the sphingomyelin-degrading SMase [19, 53]. In addition, our proteomic screening approach revealed a penicillin-binding protein (PBP) critical for cell wall modification [44, 54], a tissue-degrading collagenase [52, 55], and an alcohol dehydrogenase-like protein recently described as a pathogenic biomarker involved in *B. cereus* virulence and survival against host innate defense [56]. Overall, these findings suggest a putative role of *B. cereus* EVs in the transfer of virulence factors to host cells.

The tripartite pore-forming toxin Nhe requires the combined action of the three components NheA, NheB and NheC to induce lysis in vitro [13, 15]. Although Nhe is thought to be crucial for *B. cereus* pathogenicity, the mechanisms by which the toxin components are delivered to the host cell and assembled on host cell membranes are poorly understood. According to the current model on the mode of action of Nhe, binding of NheB and NheC occurs in solution before binding to the host cell membrane, albeit both NheB and NheC are capable of binding to cell membranes. The subsequent binding of NheA further induces pore formation and, finally, cell membrane leakage [13, 15]. NheC is assumed to be required in the priming step to induce maximum cytotoxicity [13, 14]. However, since NheC is present only in very low amounts in *B. cereus* natural culture supernatant, studies on the complex formation of Nhe components have so far only been performed with recombinant NheC in artificial systems [13–15]. It has been suggested that NheC in solution is almost 90% bound to NheB, which is necessary to induce but also to limit the toxic effect of Nhe [14]. These results from artificial systems are supported by our in vitro data using 3D-SIM microscopy, showing that NheB and NheC colocalize in *B. cereus* EVs as well as on host cell membranes.

The genes encoding the three Nhe components are organized into an operon that is polycistronic transcribed after PlcR activation [57]. Thus, the different levels of Nhe subunits usually found in supernatants of *B. cereus* cultures indicate posttranscriptional regulation and / or a regulated secretion of Nhe components. Secretion of premature Nhe components via the general secretory pathway has been described but the exact mechanism of secretion is still unknown [58]. By using highly specific antibodies for protein detection we demonstrated in our current work that NheC is strongly enriched and exclusively located in *B. cereus* EVs as compared to the vesicle-free supernatant. NheB together with NheC was detected in *B. cereus-*derived EVs, while NheA was mainly detected in the vesicle-free supernatant, supporting its role in the final stage of pore formation rather than at initiation [13, 59]. Since our data show that Nhe components are enriched in EVs, it is tempting to speculate that *B. cereus* uses EVs as an export system to transport multicomponent toxins and virulence-associated factors simultaneously and at specific concentrations to host cells. Similarly, it has been reported that *B. anthracis* uses EVs for the transport of the anthrax toxin, which comprises one binding component and two active components [44, 49]. These findings along with results from preliminary studies (data not shown) with a *B. cereus* strain producing Hbl, a further tripartite enterotoxin, foster the hypothesis that Gram-positive bacteria employ EVs as vehicles to deliver multicomponent toxins to host cells at defined concentrations and in a shielded manner.

Fusion of EVs with non-phagocytic cells via macropinocytosis, clathrin-mediated endocytosis (CME), caveolin-mediated endocytosis, and non‐caveolin/non-clathrin-mediated endocytosis (using lipid rafts or direct membrane fusion) has been well described as pathways for the uptake of outer membrane vesicles (OMVs) from Gram-negative bacteria [60]. In contrast, there is not much data available about the uptake mechanism of EVs from Gram-positive bacteria by host cells. Transcytosis of probiotic *B. subtilis* EVs through Caco2 monolayer has been recently suggested as a possible transport route of EVs across the epithelium to the bloodstream and surrounding tissue and organs [61]. As the gastrointestinal tract is essential for the virulent life cycle of *B. cereus*, we used human intestinal epithelial Caco2 as an in vitro model to study the interaction of *B. cereus*-derived EVs with the host. Consistent with previous reports in *S. aureus* [38, 48], we could show that *B. cereus* EVs fuse with human intestinal epithelial Caco2 cell membranes via cholesterol-rich domains in a time-dependent manner, which was strongly blocked by Filipin III. Besides, Dynasore, an endocytosis inhibitor, entirely blocked the uptake of *B. cereus* vesicles by Caco2 cells, implying that *B. cereus* utilizes dynamin-mediated endocytosis as an entry route. Some reduction of vesicle internalization was also observed with Chlorpromazine, an inhibitor of clathrin-dependent endocytosis. However, no effects were observed when blocking macropinocytosis and actin-dependent endocytosis. A recent study showed that *S. aureus* EVs are internalized by macrophages predominantly via the dynamin-mediated pathway whereas no effect was observed by using inhibitors for clathrin-, lipid raft-, and actin-dependent endocytosis [62]. These findings highlight the differences in the architecture and composition of EVs derived from various bacterial species while indicating the conservation of host uptake mechanisms. Both membrane fusion and endocytosis depend on the integrity of EVs, which allow direct delivery of concentrated components into host cells, enhancing thereby cell damage and immunomodulation. Although our results presented here emphasize the importance of cholesterol-rich domains and dynamin in *B. cereus* EV uptake, it could not be excluded that *B. cereus* EVs exploit diverse entry routes for their cargo delivery to diverse host cells. Thus, further studies will be needed to fully decipher the mechanism of interactions between *B. cereus-*derived EV and host cells.

Our data further demonstrated that upon successful membrane fusion, the three components of Nhe were in close proximity to the cell membrane region of human epithelial Caco2 cells. This finding is in line with previous studies reporting that NheC contains a putative hydrophobic membrane integrative region that is essential for binding to cell membranes [13, 14, 63]. In addition, Hbl component L1 and component B, which share 40% and 25% sequence identities with NheB and NheC, respectively [2], harbor putative transmembrane regions that facilitate the binding to the cell membrane, leading to rapid cell lysis or activation of the NLRP3 inflammasome in a manner dictated by the bioavailability and concentration of Hbl [64]. A similar effect might also apply to the assembly of Nhe components.

In addition to intact bacteria, uptake of bacterial EVs and their cargo by host cells can also lead to cytotoxicity, depending on the proteinaceous cargo the EVs contain. For instance, internalization of probiotic *B. subtilis* EVs by Caco2 cells did not affect cellular proliferation or viability [61], while pathogenic *S. aureus* EVs of different strains showed versatile cytotoxic potential towards host cells [47, 65]. The pathogenic potential of *B. cereus* strains also varies widely, ranging from strains that show no in vitro cytotoxic activity to strains that are highly cytotoxic [25, 66]. The use of bacterial supernatants obtained by low-speed centrifugation showed that *B. cereus* strain NVH0075-95 has the most potent toxic effects on human primary endothelial cells (HUVEC), in addition to Vero and human Caco2 epithelial cells. However, cell death occurred late at 24 h post-stimulation, suggesting the loss of mitochondrial function rather than rapid pore formation [67]. Similarly, we demonstrated that NVH0075-95-derived EVs, enriched in various virulence-associated mediators, elicited a cytotoxic effect on human Caco2 cells 24 h after stimulation, underscoring the critical role of EVs in *B. cereus* pathogenicity.

Despite the clinical importance of *B. cereus* in humans, little is known about the role of the immune system in host defense against this pathogen. Our data showed that *B. cereus* EVs are functionally active and able to induce hemolysis in human red blood cells in a concentration-dependent manner, which so far has only been shown on erythrocytes using recombinant Nhe components [68]. Furthermore, our study revealed that the presence of both Nhe and SMase in *B. cereus* EVs increased hemolysis compared to single mutant vesicles, emphasizing the importance of the interplay of virulence factors for *B. cereus* pathogenicity. A synergistic interaction of Nhe and SMase has also been described for *B. cereus* virulence in vivo using an insect model [23]. Based on our study, it is tempting to speculate that besides Nhe and SMase, multiple virulence factors packaged in *B. cereus* vesicles might act in concert to potentiate pathogenicity. Thus, the investigation of *B. cereus* vesicles might open new possibilities to study this synergism in more detail ex vivo as well as in vivo. Furthermore, we observed that *B. cereus* EVs interact with human monocytes, resulting in the systemic induction of TNF-α secretion. TNF-α is an endogenous alarm signal, which drives inflammatory responses in injury or infection to recruit other immune cells to evoke an immune-stimulatory cascade. Similarly, increased secretion of TNF-α has been reported in antigen-presenting cells upon exposure to streptococcal EVs [40, 43, 69], suggesting their immunomodulatory effect. A recent study in *S. aureus* revealed that pore-forming toxins and lipoproteins associated with EVs induced NLPR3-dependent caspase-1 activation via K + efflux and TLR2 signaling, respectively, in human macrophages which resulted in the cellular release of pro-inflammatory cytokines IL-1β and IL-18 and, finally, pyroptosis [62]. Interestingly, it was reported that a recombinant Nhe complex drives activation of the NLRP3 inflammasome by targeting the plasma membrane of host cells [63]. This observation may indicate that *B. cereus* utilizes EVs as a targeted toxin delivery system of both lipoproteins and functional toxins to induce NLRP3 activation, as recently shown for *S. aureus* [62]. However, further studies are needed to confirm this association.

## Limitation of this study

Currently, there are no minimal guidelines for the isolation of bacterial extracellular vesicles, in contrast to the minimal information for studies of eukaryotic extracellular vesicles defined in 2018 by a position paper of the International Society for Extracellular Vesicles (MISEV2018) [70]. In the present study, we thus employed a commonly used differential centrifugation approach for the isolation of *B. cereus* EVs. After the removal of bacterial cells and cell debris (including insoluble proteins), EVs were concentrated by filtration using a cutoff of 100 kDa to ensure that proteins < 100 kDa, possibly present in the supernatant most likely due to cell lysis, are not enriched in the vesicle fraction. Finally, EVs were pelleted by ultracentrifugation to remove the supernatant as well as the remaining soluble proteins. Alternatively, tangential flow filtration, size-exclusion chromatography (SEC), or density gradient centrifugation (DGC) can be considered as further purification steps for reaching high-quality EVs [51, 71]. However, each of the aforementioned methods for EV purification has its pros and cons, and further systematic studies are needed to define the most suitable protocols for the isolation of EVs for a defined bacterial species and the respective purpose of EV production. For instance, it has been shown by Hong et al. [71] that the purification of crude *E. coli* EVs by SEC or DGC did only result in the removal of a low number of potential contaminating proteins. Furthermore, it has been reported that higher purification methods can miss important EV compounds, for instant LPS in *E. coli-*derived EVs [72]. Thus, we opted for differential centrifugation to isolate crude EV preparations and did not use additional methods for further EV purifications. However, it cannot be completely ruled out that the vesicles in our current work are still contaminated with aggregated proteins. Therefore, we characterized single vesicles, by applying different approaches such as TEM, FTIR, and NTA to meet the general criteria of the MISEV2018 guidelines. In addition, the data from our proteomic studies revealed the presence of flotillin in *B. cereus* EVs (Additional file 3 and Fig. 2C). Flotillin is mentioned in MISEV2018 category 2b [70] as a marker to demonstrate the EV nature and the purity level of an EV preparation.

Since the main purpose of our proteomics approach was to screen for potential virulence factors in EVs, we performed only qualitative proteome analysis using two biological replicates. Thus, further quantitative proteomic studies of EVs as well as vesicle-free supernatants are needed to fully decipher the proteidogenous cargo of *B. cereus* EVs, including detailed quantitative information on toxins and other virulence-related factors. However, such studies require a substantially different proteomic approach, using specific labeling techniques (e.g., iTRAQ) or specific label-free techniques (e.g., Sequential Window Acquisition of all Theoretical Mass Spectra (SWATH) mass spectrometry). Therefore, they are beyond the scope of our current work, which focuses on the biological activity of *B. cereus* EVs.

## Conclusion

Though considerable progress has been made in the characterization of EVs from Gram-positive bacteria [30, 31], their role in *B. cereus* pathogenicity remained elusive. The discovery of *B. cereus* vesicles provides the first insights into the protective transfer of *B. cereus* multicomponent toxins to host cells and their assembly at host cell membranes under physiological conditions. It thus opens up new possibilities for deciphering their molecular mechanisms of action.

Our results demonstrate that EVs produced by *B. cereus* serve as a secretory pathway to deliver bacterial effector molecules to the host simultaneously and at defined concentrations, enabling their concerted and synergistic action on target cells. EVs derived from a wild-type strain and isogenic knockout mutants proved to be a valuable tool to fine-tune the EV protein cargo for studying synergistic interactions of pore-forming toxins and cell membrane active enzymes and also provide new opportunities to study immunomodulating functions of bacterial effectors delivered to the host by EVs.

## Methods

### *B. cereus* strains and growth conditions

The enteropathogenic *B. cereus* strain NVH0075-95, isolated from vegetables after a large food poisoning outbreak in Norway [7], and its isogenic mutant strains *Δsmase, ΔnheBC*, and *ΔnheBCΔsmase* [23] were routinely grown in Lysogeny Broth (LB) or on LB agar plates at 30 °C.

### Isolation of *B. cereus* EVs

EVs were isolated from *B. cereus* culture supernatants, as described previously with minor modifications [44]. Briefly, *B. cereus* strains were inoculated at a cell density of OD_600_ of 0.05 in 50 mL LB-medium and grown for 17 h at 30 °C under shaking (120 rpm). After removal of the bacterial cells at 3,000 × *g* and 4,000 × *g* for 15 min at 4 °C, the EV containing supernatant was sterile filtered (0.45 µm cutoff) and centrifuged at 10,000 × *g* for 15 min at 4 °C to remove cellular debris. Subsequently, an Amicon® ultrafiltration system (100 kDa cutoff; Millipore, USA) was used to concentrate the EVs and remove soluble proteins (< 100 kDa) and supernatant. Finally, EVs were collected by ultracentrifugation at 125,000 × *g* for 1 h at 4 °C in a TLA-45 rotor (Optima TLX centrifuge; Beckman Coulter, USA). EV pellets were washed and resuspended in phosphate-buffered saline (PBS). Protein determination was performed using DC Protein Assay (BioRad, Vienna, Austria) according to the manufacturer’s instructions. Proteins in the vesicle-free filtrate were precipitated by 10% ice-cold trichloroacetic acid (TCA) solution as reported [66]. Protein concentration from precipitated proteins was determined using the 2-D Quant Kit (GE Healthcare, USA), according to the manufacturer’s instructions.

The characterization of EVs was performed according to minimal information for studies of extracellular vesicles (MISEV) 2018 guidelines [70]. Mass spectrometry was used to determine the protein composition of EVs, whereas transmission electron microscopy (TEM) and nanoparticle tracking analysis (NTA) were utilized to visualize their characteristic lipid-bilayer structure and size, respectively. Protein, fatty acids, and polysaccharide ratios of EVs compared to the bacterium were determined by means of FTIR spectroscopy. Furthermore, FTIR spectroscopy was used to monitor the quality of EV preparations (see description in the section FTIR spectroscopy).

### Size distribution using nanoparticle tracking analysis (NTA)

The effective diameter and size distribution of EVs were measured using ZetaView × 30 TWIN Laser System 488/640 (Particle Metrix, Inning am Ammersee, Germany) as described [73, 74]. For this purpose, EVs were diluted 1:1,000 in shortly prior sterile-filtered PBS and the instrument was calibrated using 100 nm polystyrene beads. Particle tracking analysis was performed in scatter mode with a 488 nm laser with the following settings: Minimum brightness 30; minimum area 10; maximum brightness 255; maximum area 1000; temperature 25 °C; shutter of 70; sensitivity was adjusted to achieve the appropriate amount of traces, as suggested by the manufacturer.

### Fourier transform infrared (FTIR) spectroscopy

Differences in the metabolic fingerprints between bacteria and EVs as well as the robustness of EV isolations were assessed by FTIR spectroscopy. Therefore, purifying EVs from six independent bacterial cultures were subjected to FTIR spectroscopy as described previously [75]. In brief, suspensions containing either EVs or bacterial cells were prepared and transferred to zinc selenite (ZnSn) optical microtiter plates (Bruker Optics GmbH, Ettlingen, Germany) and dried at 40 °C for 40 min. FTIR spectra were recorded in transmission mode with the aid of an HTS-XT microplate adapter coupled to a Tensor 27 FTIR spectrometer (Bruker Optics GmbH, Germany) using the following parameters: 4000 to 500 cm^−1^ spectral range, 6 cm^−1^ spectral resolution, averaging of 32 interferograms with background subtraction for each spectrum.

To compare FTIR spectra derived from bacterial cells and EVs, FTIR spectra were preprocessed using vector normalization, baseline correction and calculation of second derivates over the whole spectra using a second-order 9-point Savitzky–Golay algorithm. Spectroscopic ratios of fatty acids (3020 – 2800 cm^−1^), proteins (1720—1500 cm^−1^) and polysaccharides (1200—900 cm^−1^) of bacterial cells versus EVs were calculated as described previously with minor modifications [75]. In brief, raw spectra were baseline corrected and smoothed using the Savitsky-Golay method (5 smoothing points, 3rd-grade polynomial), followed by total integration of the indicated areas, whereas the integration of the amide area was fitted using Lorentzian component bands.

### Transmission electron microscopy (TEM) analysis

For transmission electron microscopy (TEM) imaging, pelleted EVs were fixed in 3% neutral buffered glutaraldehyde (Merck Millipore, USA), pre-embedded in 1.5% agar and washed in Sorenson's phosphate buffer (pH 6,8; Morphisto, Vienna, Austria), as described previously [76]. After post-fixation in 1% osmium tetroxide (Electron Microscopy Sciences, Hatfield Township, PA, USA), samples were sequentially dehydrated in ethanol series, soaked in propylene oxide and embedded in epoxy resin (Serva Electrophoresis GmbH, Heidelberg, Germany). Ultrathin sections (70 nm) were obtained with a Leica Ultramicrotome (Leica Ultracut S, Vienna, Austria) and contrasted with alkaline-lead citrate (Merck Millipore, USA) and methanolic-uranyl acetate (Sigma-Aldrich, USA). Vesicle structures were visualized on a transmission electron microscope Zeiss EM 900 (Carl Zeiss Microscopy GmbH, Jena, Germany) equipped with a digital Frame-Transfer-CCD camera (Tröndle TRS, Moorenweis, Germany).

### Liquid chromatography-tandem mass spectrometry (LC–MS/MS)

#### Sample preparation and digestion by filter aided sample preparation (FASP)

Isolated *B. cereus* EVs were prepared for proteomic analysis to screen for virulence factors as described previously [77]. Equal protein amounts of isolated *B. cereus* EVs were precipitated with 10% ice-cold TCA [78, 79]. Precipitated proteins were washed with ice-cold acetone, air-dried and re-suspended in 6 M urea, 2 M thiourea and 10 mM TRIS. In total, 30 µg of protein was mixed with 8 M Urea in 50 mM TRIS and loaded onto an Amicon 10 kDa filter (2 × 20 min at 10,000 × *g*). The samples were reduced with 200 mM dithiothreitol (DTT) (37 °C, 30 min) and alkylated with 500 mM iodoacetamide (IAA) (37 °C, 30 min). After washing with 50 mM TRIS twice, digestion was done using Trypsin/LysC Mix in a ratio of 1:25 (protease:protein) overnight at 37 °C. Digested peptides were recovered in 150 µl of 50 mM TRIS and acidified with 0.1% trifluoroacetic acid (TFA). Prior to LC–MS analysis, peptide extracts were desalted and cleaned using C18 spin columns (Pierce Biotechnology, USA) according to the manufacturer’s protocol. The dried peptides were dissolved in 300 µl 0.1% TFA, of which 3 µl were injected into the LC–MS/MS system.

#### Mass spectrometry and data analysis

Peptides were separated and identified on a nano-HPLC Ultimate 3000 RSLC system (Dionex, USA) coupled to a high-resolution Q Exactive HF Orbitrap mass spectrometer (Thermo Fisher Scientific, USA). Raw spectra were subjected to database searches using Proteome Discoverer Software 2.4.0.305 with the Sequest HT algorithm (Thermo Fisher Scientific, USA). The UniProt database for *B. cereus* (taxonomy 1396, accessed on 11.5.2022) and a common contaminant database (https://www.thegpm.org/crap/; accessed on 11.5.2022) were used for query of the spectra. Following search parameters were applied: Trypsin as an enzyme with a maximum of two allowed missed cleavages; 10 ppm precursor mass tolerance and 0.02 Da fragment mass tolerance. As dynamic modifications oxidation/ + 15.955 Da (M) and deamidation/ + 0.984 Da (N, Q), as N-terminal modifications acetyl/ + 42.001 Da (N-Terminus), Met-loss/-131.040 Da (M) and Met-loss + Acetyl/89.030 Da (M) and as fixed modifications Carbamidomethyl/ + 57.021 Da (C) was used. Proteins were identified on the basis of at least two peptides and strict false discovery rate targets of 0.01 (1%) threshold in all nodes in Proteome Discoverer 2.4.0.305 (Thermo Fisher Scientific). The overlap of protein identities in the biological replicates was used for further analysis. Signal peptide cleavage sites were predicted using SignalP v6.0 [80], subcellular locations were predicted using PsortB v3.0.3 (https://www.psort.org/psortb/ and gene-term enrichment analysis was performed using KEGG and by setting a false discovery rate (FDR) to < 0.05.

### Immunoblotting

Proteins from EVs and TCA-precipitated EV-free supernatants were separated on 12.5% SDS-PAGE gels and blotted on a nitrocellulose membrane (BioRad Transblot SD Semi Dry Transfer Cell, BioRad, Vienna, Austria). Subsequently, blotted proteins were labeled either with mouse monoclonal anti-SMase antibody (0.5 µg/mL, 33kDaI-MAb 2A12), mouse monoclonal antibodies anti-NheA IgG1 (1A8; 2.5 µg/mL), anti-NheB IgG1κ (1E11; 1.25 µg/mL), and anti-NheC IgM (3D6; 5 µg/mL) [81] followed by peroxidase-conjugated goat anti-mouse IgG antibody (1:20,000) (Dianova, Hamburg, Germany). Generally, 5 µg of total protein was used for immunoblotting. In addition, 40 µg total protein of EV-free supernatants was tested for NheC. Immunoreactive bands were visualized using Super Signal West Pico Chemiluminescent Substrate (Thermo Fisher Scientific, USA) and scanned with ChemiDoc MP Imaging System (BioRad, Vienna, Austria).

### EV uptake assay

Uptake of EVs by human intestinal Caco2 epithelial cells was monitored using the self-quenching lipophilic dye Octadecyl Rhodamine B Chloride (R18; Molecular Probes, Life Technologies, USA) as described [82, 83]. In brief, 5 µg of EV protein were stained with 1 mg/mL R18 for 1 h at room temperature, followed by two washing steps in 0.2 M NaCl at 125,000 × *g* for 1 h at 4 °C. Prior to vesicle uptake, 1 × 10^4^ Caco2 cells were incubated in MEM/10% FBS for 48 h in 96-well plates (Corning Inc., USA) at 37 °C in 5% CO_2_ atmosphere and were then incubated with R18-labeld EVs in 100 µL 0.2 M NaCl per well. To inhibit vesicle uptake, Caco2 cells were treated either with cholesterol-sequestering agents Filipin III (10 ug/ml) and Imipramine (10 mM), or endocytosis inhibitors Dynasore (80 μM), Cytochalasin D (1 μg/ml), Chlorpromazine (15 ug/ml), and Amiloride (10 mM; all from Sigma Aldrich). The substances were added 1 h prior to the addition of R18-labeled vesicles. Fluorescence was detected every 2 min for a total period of 90 min at 37 °C with a fluorescence reader (570/595 nm; SpectraMax M3, Molecular Devices, USA). The % of EV uptake was calculated after 90 min of treatment and normalized to untreated control.

### Cytotoxicity assay

Cytotoxicity of EVs was quantified using a cell culture assay based on Caco2 cells. Caco2 cells (2 × 10^5^ cells/mL) were incubated with 200 µg/mL EVs in MEM-Earle medium supplemented with 2% fetal calf serum (FCS; v/v) for 24 h at 37 °C in a 5% CO_2_ atmosphere. The viability of the cells was measured using the Vita-Orange Cell Viability Reagent (Biotool, Switzerland), according to the manufacturer’s protocol. The viability of treated cells was determined as a percentage compared to untreated control cells.

### Super-resolution microscopy

#### Visualization of Nhe containing extracellular vesicles

Extracellular vesicles (25 µg) were fixed with 4% paraformaldehyde (PFA) and incubated consecutively with mouse monoclonal antibody NheA IgG1 (1A8; 12.5 µg/mL), NheB IgG1k (1E11; 12.5 µg/mL) and NheC IgM (3D6; 17.5 µg/mL) for 1 h. After washing steps with PBS/5% BSA, Nhe proteins were labeled with secondary goat anti-mouse IgG AlexaFluor®488 and goat anti-Mouse AF568 IgM (4 µg/mL, Molecular Probes, Life Technologies, USA).

#### Delivery of Nhe-containing EVs to Caco2 cells

Caco2 cells were cultured in 8-well ibidi µ-slides (ibidi GmbH, Martinsried, Germany) and treated with 200 µg/mL of EVs for 2 h at 37 °C, 5% CO_2_. After washing with PBS, cells were fixed with 4% PFA and incubated with a buffer solution containing 0.05% bovine serum albumin (BSA), 0.1% Triton X-100, and 0.025% Tween 20 in PBS. Cells were incubated consecutively with mouse monoclonal antibody NheA IgG (1A8) and NheC IgM (3D6) or NheB IgG1κ (1E11) and NheC IgM (3D6) for 1 h at a final concentration of 4 µg per well and were detected with the secondary goat anti-mouse IgG AlexaFluor®488 and goat anti-Mouse AF568 IgM (2 µg per well, respectively) (Molecular Probes, Life Technologies, USA). Nuclei were stained with 1.5 μM 4',6-diamidino-2-phenylindole (DAPI, Sigma Aldrich). Fluorescence 3D-SIM (structured illumination microscopy) images were acquired with the Zeiss LSM710 Elyra PS.1 microscope system equipped with an Andor iXon 897 (EMCCD) camera. Image processing was performed using the Zeiss ZEN 2012 software (Carl Zeiss Microscopy GmbH, Jena, Germany).

### Hemolysis assay

Human erythrocytes from three different donors were isolated from leukocyte reduction system (LRS) chambers of a Trima Accel® automated blood collection system (Terumo BCT, USA) by Ficoll gradient centrifugation. Plasma and mononuclear cell layer were removed and erythrocytes were washed twice with PBS (pH 7.4). A quantitative hemolytic assay was performed as described earlier [82]. Briefly, 50 µL human erythrocytes (5 × 10^8^/mL) were incubated with an equal volume of 12.5, 25 and 50 µg of EVs for 1 h at 37 °C. PBS and 5% (v/v) Triton X-100 served as negative and positive controls, respectively. After incubation, 100 µL ice-cold PBS was added and centrifuged at 400 × *g* for 15 min at 4 °C. The hemolytic activity from the supernatant was determined by the release of hemoglobin at 540 nm (SpectraMax M3, Molecular Devices, USA) and calculated as percentage relative to the positive control.

### Isolation and stimulation of human primary monocytes with EVs

Human peripheral blood mononuclear cells (PBMCs) from three different donors were isolated from leukocyte reduction system (LRS) chambers of a Trima Accel® automated blood collection system (Terumo BCT, USA) and cultured as previously described [84]. To characterize the pro-inflammatory potential of EVs, 200 µl of human primary monocytes (2.5 × 10^6^/mL) were stimulated with 100 µg/mL of EVs for 4 h in a 96-well plate at 37 °C in RPMI medium. Supernatants were collected and the concentration of TNF-α was quantified by ELISA (Merck Millipore, USA).

### Statistical analysis of data

Statistical analyses for at least three independent biological replicates were performed with the aid of GraphPad Prism8 software (GraphPad Software, Inc., USA). Statistical significance was calculated using Student’s two-tailed unpaired t-test and the two-way ANOVA with Tukey's multiple comparisons test was applied for multiple comparisons. Statistical significance was concluded when a probability value (*p* value) was lower than 0.05.

### Supplementary Information

**Additional file 1: Figure S1.**

**Additional file 2: Figure S2.**

**Additional file 3: Table 1.**

**Additional file 4: Table 2.**

**Additional file 5: Table 3.**

## Data Availability

The datasets used and/or analyzed in the present study are available from the corresponding author on reasonable request. The mass spectrometry proteomics data have been deposited to the ProteomeXchange Consortium via the PRIDE [85] partner repository with the dataset identifier PXD041561.

## Abbreviations

ABC: ATP-binding cassette
Caco2: Human colon carcinoma cell line 2
CME: Clathrin-mediated endocytosis
DTT: Dithiothreitol
EVs: Extracellular vesicles
FASP: Filter aided sample preparation
FCS: Fetal calf serum
FDR: False discovery rate
FTIR: Fourier tansform infrared spectroscopy
Hbl: Hemolysin BL
IAA: Iodacetamide
LB: Lysogeny broth
LC–MS/MS: Liquid Chromatography-Tandem Mass Spectrometry
LRS: Leukocyte reduction system
MISEV: Minimal information for studies of erxtracellular vesicles
Nhe: Non-hemolytic enterotoxin
NTA: Nanoparticle tracking analysis
OMVs: Outer membrane vesicles
PBMCs: Peripheral blood mononuclear cells
PBP: Penicillin binding protein
PBS: Phosphate-buffered saline
PFA: Paraformaldehyde
PLC: Phospholipase C
R18: Octadecyl rhodamine B chloride
SMase: Sphingomyelinase
TCA: Thrichloroacetic acid
TEM: Transmission electron microscopy
TFA: Trifluoroacetic
TNF-α: Tumor necrosis factor-alpha
