# Correction: *Bacillus cereus* extracellular vesicles act as shuttles for biologically active multicomponent enterotoxins

**DOI:** 10.1186/s12964-024-01477-1

**Published:** 2024-01-15

**Authors:** Tanja Buchacher, Astrid Digruber, Markus Kranzler, Giorgia Del Favero, Monika Ehling-Schulz

**Affiliations:** 1 Functional Microbiology, Department of Pathobiology, Institute of Microbiology, University of Veterinary Medicine, Vienna, Austria; 2 Department of Food Chemistry and Toxicology, Faculty of Chemistry, University of Vienna, Vienna, Austria; 3 Core Facility Multimodal Imaging, Faculty of Chemistry, University of Vienna, Vienna, Austria

**Correction: Cell Commun Signal 21, 112 (2023)**

**https://doi.org/10.1186/s12964-023-01132-1**

Following publication of the original article [1], the authors identified an error in the author name of Markus Kranzler.

The incorrect author name is: Markus Kanzler

The correct author name is: Markus Kranzler

The author group has been updated above and the original article [1] has been corrected.
