# Supplementary material for: Bacillus cereus extracellular vesicles act as shuttles for biologically active multicomponent enterotoxins
Source: Cell Commun Signal. 2023 May 15;21:112. doi: 10.1186/s12964-023-01132-1 (PMC10184354; doi:10.1186/s12964-023-01132-1)
Supplement: Supplementary file 2 — Additional file 1: Figure S1. [file 12964_2023_1132_MOESM1_ESM.pdf]

Additional file 1: Figure S1

A

GO term ID	Molecular function	FDR
GO:0003824	Catalytic activity	5.98e-08
GO:0046872	Metal ion binding	0.00016
GO:0016491	Oxidoreductase activity	0.0014
GO:0016624	Oxidoreductase activity, acting on the aldehyde or oxo group of donors, disulfide as acceptor	0.0151
GO:0016903	Oxidoreductase activity, acting on the aldehyde or oxo group of donors	0.0218
GO:0016829	Lyase activity	0.0327
GO:0008237	Metallopeptidase activity	0.0341
GO:0016787	Hydrolase activity	0.0341
GO:0046914	Transition metal ion binding	0.0390
GO:0016668	Oxidoreductase activity, acting on a sulfur group of donors, nad(p) as acceptor	0.0400
GO:0043167	Ion binding	0.0400

B

GO term ID	Cellular component	FDR
GO:0005576	Extracellular region	2.90e-16
GO:0110165	Cellular anatomical entity	2.41e-06
GO:0030312	External encapsulating structure	9.79e-06
GO:0005618	Cell wall	0.00035
GO:0030288	Outer membrane-bounded periplasmic space	0.0034
GO:0005622	Intracellular	0.0057
GO:0005737	Cytoplasm	0.0057
GO:0009288	Bacterial-type flagellum	0.0065
GO:0042597	Periplasmic space	0.0065
GO:0009424	Bacterial-type flagellum hook	0.0124
GO:0043190	ATP-binding cassette (ABC) transporter complex	0.0124
GO:0098796	Membrane protein complex	0.0124
GO:0030115	S-layer	0.0298

Figure S1 (A-B) Gene ontology (GO) enrichment analysis was performed on identified proteins. The top significantly enriched GO terms for (A) molecular function and (B) cellular component are shown (FDR < 0.05).
