# Supplementary material for: Bacillus cereus extracellular vesicles act as shuttles for biologically active multicomponent enterotoxins
Source: Cell Commun Signal. 2023 May 15;21:112. doi: 10.1186/s12964-023-01132-1 (PMC10184354; doi:10.1186/s12964-023-01132-1)
Supplement: Supplementary file 3 — Additional file 2: Figure S2. [file 12964_2023_1132_MOESM2_ESM.pdf]

Additional file 2: Figure S2

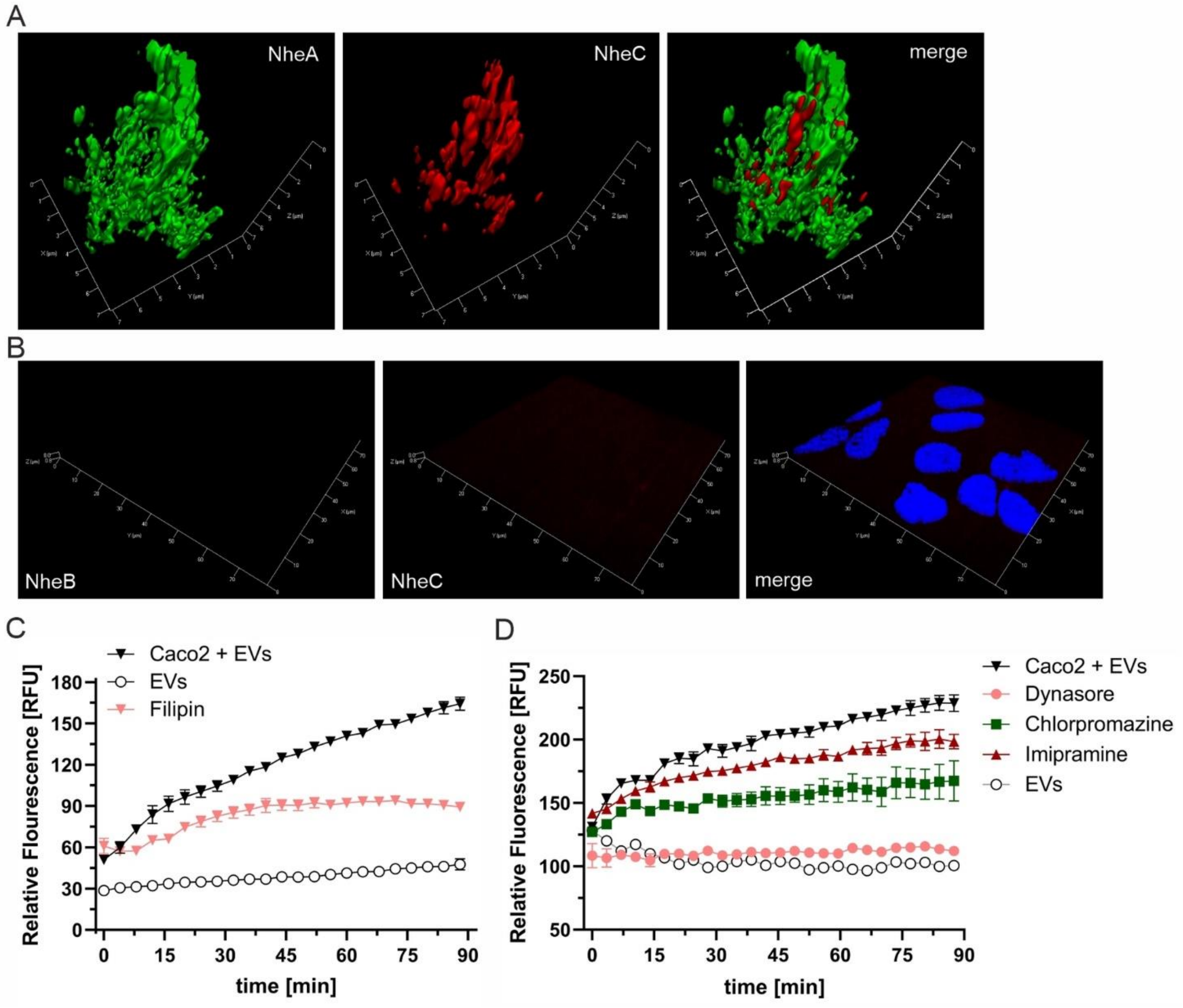

Figure S2 (A) The presence of NheA (AF488, green) and NheC (AF568, red) in *B. cereus* vesicle aggregates were confirmed with 3D-SIM-microscopy. (B) As a control, Caco2 cells without EV treatment were stained with anti-NheB (AF488, green), anti-NheC (AF568, red) and cell nuclei (DAPI; blue) and visualized by 3D-SIM-microscopy. (C-D) Inhibition of vesicle uptake by Caco2 was studied in the presence of either (C) cholesterol-sequestering agents Filipin III (10 µug/ml) (D) and Imipramine (10 mM), or dynamin and clathrin-mediated endocytosis inhibitors Dynasore (80 µM), and chlorpromazine (15 ug/ml; all from Sigma Aldrich, USA), respectively. After 1 h of cell treatment with the inhibitors, Rhodamine-R18 labeled *B. cereus* vesicles (5 µg) EVs were added and cultured for 90 min. The fluorescence intensity was measured every two minutes (shown here every six minutes) up to 90 min at 37°C using a microtiter reader. Increase in fluorescence intensity indicates membrane fusion.
